# Retraction: The role of trade liberalization in promoting regional integration and sustainability: The case of regional comprehensive economic partnership

**DOI:** 10.1371/journal.pone.0286355

**Published:** 2023-05-23

**Authors:** The PLOS ONE Editors

This article [1] reported the use of RunGTAP software and 2014 data from the GTAP 10 database.

Following the publication of this article [1], concerns were raised that the authors of this study did not obtain a formal licence to use the GTAP10 database reportedly used in this study, and that instead access to the database was obtained through an unofficial channel.

PLOS followed up with the corresponding author regarding these concerns, who stated that the RunGTAP software used does not require a license to run aggregations of less than 10 by 10; however, they did not provide a copy of the license required to use the GTAP 10 database to PLOS for editorial review, indicating difficulties locating the relevant information. PLOS followed up with the Center for Global Trade Analysis who confirmed that the RunGTAP software licensing is separate from the GTAP database licensing, a GTAP 10 database is not yet publicly available, and a valid user license is required to analyse data from this database using the RunGTAP software [2, 3, 4].

In light of the absence of proof of a user license from the Center for Global Trade Analysis required for the use of the GTAP 10 database for this study and to publish the dataset and results reported in this article, the *PLOS ONE* editors retract this article [1].

Owing to the above concerns, the retracted *PLOS ONE* article was removed from the *PLOS ONE* website at the time of retraction. The article’s Copyright and Data Availability statements were updated at the time of retraction and removal, and the removed contents are no longer offered under the Creative Commons Attribution License.

YW and YuChen agreed with the retraction. ZJ did not respond to the editorial decision. YiChen either did not respond directly or could not be reached.
